# The Müller-Lyer illusion as seen by an artificial neural network

**DOI:** 10.3389/fncom.2015.00021

**Published:** 2015-02-19

**Authors:** Otto B. García-Garibay, Victor de Lafuente

**Affiliations:** Department of Developmental Neurobiology and Neurophysiology, Instituto de Neurobiología, Universidad Nacional Autónoma de MéxicoQuerétaro, México

**Keywords:** Müller-Lyer, HMAX, visual illusion, visual system, perception

Visual illusions are sensory percepts that can't be explained completely from the observed image but that arise from the internal workings of the visual system. In them, we perceive something that is not physically present in the image, and are of interest to neuroscientists because they reveal visual processing that we are not normally aware of. For example, the simultaneous contrast illusion lets us appreciate that we do not perceive luminance in absolute values and that, instead, the visual system calculates an object's luminance in relation to its surroundings (Figure 1A).

**Figure 1:**
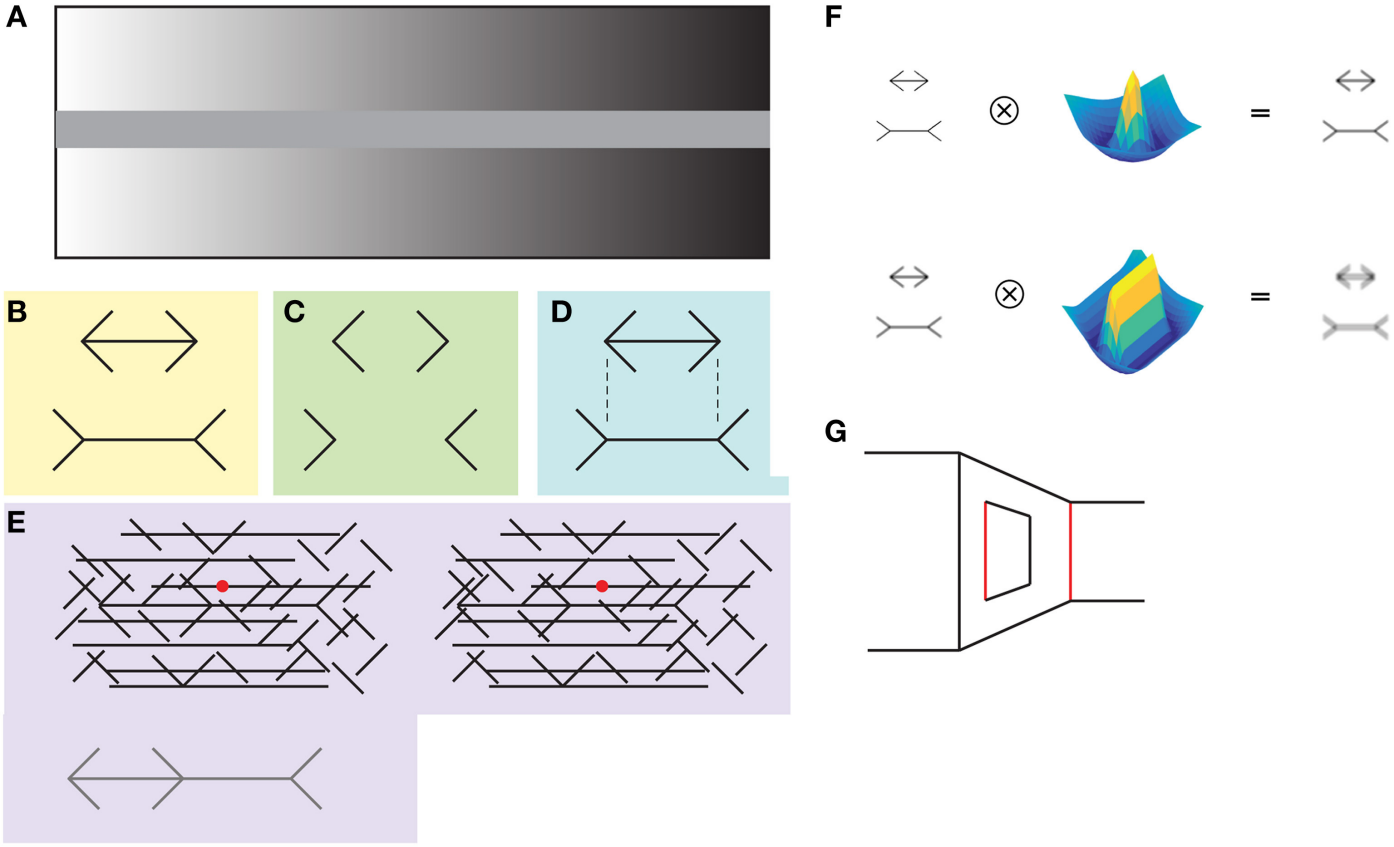
**(A) In the simultaneous contrast illusion the uniformly gray central bar appears more luminant at the right, when a dark background surrounds it**. **(B)** In the classical form of the Müller-Lyer illusion, the horizontal line with arrowheads looks shorter than the horizontal line with arrowtails. **(C)** The illusion is also present without the horizontal lines. **(D)** Notice that the illusion is not present when the viewer analyzes local features, for example, by determining whether the vertices are aligned vertically. It can be appreciated that the vertices are aligned vertically **(B)**, even though this perception contradicts the illusory effect of the horizontal lines having different lengths. **(E)** The contiguous version of the illusion (depicted at the bottom) is hidden within a background of lines. The Müller-Lyer figure appears above the background when both images are fused by decreasing eye vergence, i.e., as if focusing an object behind the plane of the image. **(F)** The low-level explanation states that the illusion arises from the low-pass properties of center-surround (upper panel) and simple cells (lower panel) at earlier stages of the visual processing. This hypothesis was not favored by the results of Zeman and colleagues. **(G)** The “carpentered world” explanation states that arrowheads and tails indicate that the lines are corners at different depths and that the visual system calculates the size of the lines taking this into account. The red lines have the same length.

By dissociating our sensory percepts from the physical characteristics of a stimulus, visual illusions provide neuroscientists with a unique opportunity to study the neuronal mechanisms underlying our sensory experiences (Eagleman, 2001; Panagiotaropoulos et al., 2012). The salient percepts that visual illusions create, along with the fact that they arise from internal processing, constantly stimulates researchers to search for the mechanism and the location within the brain where illusions originate. However, illusions have proven as difficult to explain as any other perceptual phenomena.

The physiological origins of some illusions have been investigated in animals, some of which are known to perceive them similarly to humans (Tudusciuc and Nieder, 2010). This research shows that perceptual phenomena such as visual masking, flash suppression, filling-in, motion-induced depth, and cyclopean perception (random dot stereograms) are present in early stages of the visual processing in structures such as the thalamus, and the primary and secondary visual cortices (Carney et al., 1989; Macknik et al., 2000; von der Heydt et al., 2000; Grinvald and Hildesheim, 2004; Wilke et al., 2009).

The Müller-Lyer illusion (MLI) is a simple and much studied geometrical illusion that in its classical form consists of two horizontal line segments that are perceived to have different lengths depending on whether they have arrowheads or arrowtails at their endpoints (Figures 1B–E). In an effort to understand the neuronal mechanisms behind the illusion previous work by Zeman et al. (2013) demonstrated that the MLI is present in the multi-layered artificial network HMAX, which is a model that incorporates many features of the primate visual system (Serre et al., 2005). The authors first trained the network to categorize images of short and long horizontal shafts, presented in configurations that do not evoke the illusion in humans. After this training they asked the network to classify the shaft lengths of images containing the classical MLI.

The results show that the HMAX network showed a bias in the classification of the horizontal shafts, classifying the ones with arrowheads as shorter than actually were. Interestingly, the magnitude of the bias was similar to that measured in humans, and this effect was also modulated by the angle of the fins, with smaller angles (closer to the horizontal shaft) producing a larger bias. Importantly, the authors demonstrated that the final classification layer, i.e., the layer that categorizes the images as long or short, does not rely only on units with high spatial frequencies. This result fails to support the low-level explanation of the illusion stating the low-pass characteristics of the center-surround and simple cells might be the principal cause of the illusion (Figure 1F). Furthermore, given that the network was not trained with natural images, and that it did not contain information relative to depth, the high-level “carpentered world” explanation of the illusion was not favored either (Figure 1G; Segall et al., 1963; Ninio, 2014).

The new work of Zeman et al. (2014) elaborates those previous results by demonstrating that the magnitude of the illusion increases after processing by layers of simple cells, and that it decreases after processing by layers of complex cells. The reduction of the illusion by complex cells suggests that the property of positional invariance (the ability to respond to a stimulus despite its spatial location) could make those neurons less sensitive to the bias induced by the illusion. These new results indicate that the magnitude of the MLI might be represented differently across different neuronal populations, and that more abstract representations of the images might be less sensitive to the illusory effects.

The mechanisms behind the illusion are still elusive. As Zeman and colleagues show, the low-level explanation, despite its attractive simplicity, might not be the complete story. As has been shown with random dots stereograms and other binocular versions of the illusion (Figure 1E), the MLI can be generated at a processing level beyond those of simple center-surround receptive fields, even in the absence of luminance contrast (Julesz, 1971). Although the “carpentered world” hypothesis is not necessary to explain the illusion, the involvement of the parietal and occipito-temporal cortices suggest that it is likely that higher cognitive processes are involved (Weidner and Fink, 2007; Mancini et al., 2011).

The MLI demonstrates that the intuitively simple instruction “compare the length of the two horizontal lines” is not carried by the visual system as straightforwardly as it subjectively feels. It is clear that the visual system is comparing something else across the drawings, and it might be related to complete visual objects, not to local information. When asked about the size, our visual system might be judging the size of the complete objects. This can be demonstrated by focusing our attention into a local feature of the Müller-Lyer drawing, for example by trying to determine whether the endpoints of the arrows are aligned vertically (Figure 1D). It can be appreciated, even in Figure 1B or Figure 1C, that vertices are aligned vertically, a perception that indicates the illusion is not present at a local level.

The MLI illusion is a deceptively simple perceptual experience that keeps attracting the attention of neuroscientists. The work of Zeman and colleagues suggests that two often cited causes of the illusion, the low pass filtering properties of visual neurons and the “carpentered world” hypothesis, are not needed to generate the illusion within a primate-like visual system. Future work will be needed to elucidate the mechanisms by which the brain estimates and compares the size of visually identified objects.

## Conflict of interest statement

The authors declare that the research was conducted in the absence of any commercial or financial relationships that could be construed as a potential conflict of interest.
